# The relationship between auditory steady-state response and behavioural audiometry in hearing estimation for infants: a meta-analysis

**DOI:** 10.1186/s13643-025-03003-x

**Published:** 2025-12-03

**Authors:** Xin Huang, Karolina Kluk, Emanuele Perugia

**Affiliations:** Manchester Centre for Audiology and Deafness (ManCAD), School of Health Sciences, Faculty of Biology, Medicine and Health, University of Manchester, Manchester, UK

**Keywords:** Auditory Steady-State Response, Behavioural Audiometry, Newborn Hearing Screening, Meta-Analysis, Threshold Difference

## Abstract

**Background:**

Auditory Steady-State Response (ASSR) allows the identification of infants with hearing loss and consequently early intervention. Therefore, it is important to assess the accuracy of ASSR for determining hearing thresholds in infants. This study aimed to systematically review the threshold differences between air-conducted ASSR and behavioural audiometry (BA) in infants.

**Methods:**

The population was infants younger than 2 years; the intervention was ASSR thresholds; the comparator was BA thresholds; and the outcome was the ASSR-BA threshold, i.e., the correction value in dB. PubMed, Web of Science, The Cochrane Central Register of Controlled Trials, and Embase were searched. The risk of bias was evaluated using the Evidence Project risk of bias tool and the Newcastle–Ottawa Quality Assessment Scale. The mean and 95% confidence intervals (CI) were calculated for the threshold differences at four frequencies (0.5, 1, 2, and 4 kHz).

**Results:**

Of 503 articles identified, 10 were eligible for a narrative summary, and seven were included in the meta-analysis, which had a total of 2845 ASSR-BA thresholds. The articles were of moderate quality and showed substantial heterogeneity (I^2^ between 89 and 96%, *p* < 0.01). The mean differences (± 95% CI) between ASSR thresholds and BA hearing thresholds were 9.24 dB (± 6.45), 7.19 dB (± 4.02), 6.35 dB (± 4.51), and 7.42 dB (± 5.40) at 0.5, 1, 2, and 4 kHz, respectively.

**Conclusions:**

ASSR provides a reasonably accurate prediction of BA thresholds. Given the heterogeneity of the included studies, further research with larger infant populations is needed.

**Systematic review registration:**

The study was pre-registered in PROSPERO and followed PRISMA-P 2015 statement.

**Supplementary Information:**

The online version contains supplementary material available at 10.1186/s13643-025-03003-x.

## Introduction

Early identification of hearing loss is crucial for the healthy development of infants and provides important information for early intervention to enable optimal hearing and language development [1]. Infants who are referred from the neonatal hearing screening are often too young (a few weeks/months old) for reliable hearing threshold assessment using standard behavioural audiometry (BA) [2]. In addition, infants with developmental delays or impairments are also not able to cooperate in the behavioural hearing test. Electrophysiological hearing tests like auditory brainstem response (ABR) or auditory steady-state response (ASSR) provide an alternative way of obtaining hearing information for these infants [3].

Tone-burst ABR is the gold-standard for estimating hearing thresholds in infants as recommended by the Joint Committee on Infant Hearing [4]. In contrast to ABR, ASSR can be used to measure severe-to-profound hearing loss (the upper limit of ASSR is at 120 dB HL) [5] and can be recorded at multiple frequencies in both ears simultaneously, potentially reducing testing time required in the clinic [6, 7].

ASSRs are envelope-following electrophysiological potentials recorded from the scalp of a patient and are evoked by rapidly changing auditory stimuli [8]. The introduction of 40-Hz event-related potential (ERP) initiated extensive research into ASSR [9]. It was shown that although the 40 Hz-ERP could be successfully recorded at sound levels close to hearing thresholds, the amplitude of the response was easily affected by sleep, making it difficult to be reliably used in infants [9, 10]. It was observed that the brainstem was the main generator source for modulation rates higher than 50 Hz, whilst brainstem and auditory cortices were the sources for lower modulation rates (≤ 40 Hz) [11, 12]. Furthermore, the amplitude of responses recorded at higher modulation rates (> 70 Hz) was found to be relatively unaffected by sleep [13]. Therefore, the high-modulation rate ASSR (70–110 Hz) was chosen as a good measure of hearing thresholds in infants [2, 14–25].

The correlation coefficients between ASSR thresholds and behavioural hearing thresholds in infants range from 0.75 to 0.98 [14, 23, 24], making ASSR a good measure for reliably estimating frequency-specific hearing thresholds in infants to support early intervention. An accurate threshold estimation relies on precise and reliable “correction” values that allow the prediction of behavioural hearing thresholds from the measured electrophysiological values, this means that once the ASSR thresholds of the infants are obtained, the behavioural hearing thresholds can be estimated by subtracting the “correction” values from the ASSR thresholds. However, the mean threshold differences between ASSR and BA in infants (ASSR thresholds minus behavioural hearing thresholds) vary between studies [2, 15, 20, 21, 23–25].

In 2003, Herdman & Stapells [26] conducted a meta-analysis (their Table 4) of threshold differences between ASSR and BA (seven studies) and found that the threshold differences were up to 10 dB, i.e., the "correction" values (mean ± standard error) were 10 dB ± 0.6 at 0.5 kHz, 6 dB ± 0.5 at 1 kHz, 7 dB ± 0.7 at 2 kHz, and 7 dB ± 0.9 at 4 kHz [26]. However, the age range of subjects included in their meta-analysis was between 1 month and 86 years [26], which can be problematic as ASSR may be affected by age [27, 28]. Later, Tlumak et al. (2007) [29] carried out another meta-analysis of ASSR hearing threshold estimation, this time for subjects older than 6 years and found threshold differences between ASSR and BA to be between 10–20 dB. However, hitherto there has not been a meta-analysis of threshold differences between ASSR and BA in infants only. Therefore, a meta-analysis is needed to determine the “correction” value between ASSR and BA thresholds in infants to improve the diagnostic accuracy of ASSR.

The goal of this systematic review and meta-analysis was to review the threshold differences between air-conducted ASSR and BA and to define the frequency-specific “correction” values for air-conducted ASSRs to enable accurate estimation of behavioural hearing thresholds in infants (younger than 2 years old). This systematic review synthesizes findings on threshold differences between ASSR and BA in infants and provides “correction” values for ASSR thresholds to estimate behavioural hearing thresholds in infants under 2 years of age.

## Methods

A systematic review with meta-analysis was conducted to establish threshold differences between ASSR and BA. The protocol of this systematic review with meta-analysis is registered in the International Prospective Register of Systematic Reviews (PROSPERO; registration number CRD42020178380). This study was conducted in accordance with the PRISMA-P 2015 statement [30]. Although PRISMA-P 2015 is intended primarily for the preparation of protocols of systematic reviews and meta-analyses, we referenced it to guide the reporting structure of our systematic review and ensure transparency and completeness in documenting our methodology, as this study was started before PRISMA 2020 was published [31, 32].

### Data sources and search strategy

A systematic literature search regarding the relationship between air-conducted ASSR and BA in hearing threshold estimation for infants was performed in PubMed, Web of Science, The Cochrane Central Register of Controlled Trials (CENTRAL), and Embase. The search term included four subject headings (infant, auditory steady-state response, audiometry, auditory threshold) and their synonyms based on Embase subject headings (Emtree) [33] and Medical Subject Headings [34]. The search and selection were conducted by the first author alone and these were checked by a second independent reviewer (one of the co-authors). Searches were conducted in these four databases up until 7 July 2020, and an updated search was performed between 12 and 14 August 2025, using the same strategy as in the previous search and restricting the minimum publication year to July 2020. A total of 503 results were obtained. After removing duplicates, 338 articles remained.

Potential articles were excluded for one or more of the following reasons: articles not written in English or published before 2000; entries with no available full text; animal studies and studies conducted on adults or children older than 2 years old; the topic of studies not relevant to ASSR or hearing estimation; studies lacking the comparison of ASSR thresholds with behavioural hearing thresholds. The reason for excluding articles published before 2000 was that this year is generally considered the beginning of the availability of commercial ASSR devices [35]. A total of 29 full texts were left to be manually reviewed. These potentially eligible articles were assessed for eligibility by two reviewers and disagreements were solved by consensus.

### Inclusion and exclusion criteria

Using the Population, Interventions, Comparators and Outcomes (PICO) framework [36], the population was defined as infants younger than 2 years; the intervention was ASSR thresholds; the comparator was BA thresholds; and the outcome was the ASSR-BA threshold, i.e., the correction value in dB. Therefore, studies had to meet all the following criteria to be included in the meta-analysis: subjects in the study should be infants who were younger than 2 years old; ASSR was used to estimate the hearing thresholds; hearing threshold estimation was compared between ASSR and BA; reported mean threshold differences between ASSR and BA (ASSR thresholds – behavioural hearing thresholds) or data which could support this calculation.

Articles were excluded if: studies included participants older than 2 years old; studies did not report mean threshold differences between ASSR and BA; articles did not pertain to research studies, i.e., reviews.

### Assessment of bias

After the process of inclusion and exclusion, 10 papers were selected and all of these were cohort studies. The risk of bias was evaluated using two tools: the Evidence Project risk of bias tool [37], and the Newcastle–Ottawa Quality Assessment Scale for cohort studies [38]. The Evidence Project risk of bias tool evaluates the following scale items: study design (cohort; control or comparison group, pre-post intervention data); participant representativeness (random assignment of participants to the intervention; random selection of participants for assessment; follow-up rate of 80% or more); equivalence of comparison groups (comparison groups equivalent on socio-demographics; comparison groups equivalent at baseline on outcome measures). Each of these are rated as being present (yes), or absent (no), or not applicable or not reported when assessing studies [37]. The Newcastle–Ottawa tool assesses the cohort study from three perspectives: selection, comparability, and outcome [38]. The quality of the studies was assessed independently by two researchers, and any differences were resolved by consensus.

### Data extraction

Data extracted were: 1) the names of the authors; 2) year of publication; 3) type of study; 4) sample size; 5) age; 6) the number of female and male participants; 7) hearing condition (with or without hearing loss); 8) stimulation parameters of ASSR; 9) the type of behavioural audiometry; 10) mean threshold differences between ASSR and behavioural audiometry at 0.5, 1, 2, 4 kHz; 11) the number of ears used to compare the thresholds between ASSR and BA at each frequency. Individual ASSR and behavioural hearing threshold values for each child and frequency were attempted to be obtained by directly contacting the authors. Three authors provided their raw data [15, 20, 23]. Data were extracted by the first author and checked in full by a second independent reviewer (one of the co-authors).

### Data analysis

Statistical heterogeneity between studies was tested using the Cochran’s Q test and the inconsistency was tested using the I^2^ test. A value of p < 0.10 was considered statistically significant and a value of I^2^ > 75% was considered to show substantial heterogeneity for non-randomised trials [39]. The means and standard deviations of threshold differences between ASSR and BA, and the number of hearing thresholds at each frequency recorded from included studies were tabulated and the following measures were calculated: the overall mean of threshold differences between ASSR and BA weighted by the inverse of the variance of the effect estimate; standard deviation; standard error; 95% confidence interval (CI). Analyses were run in Review Manager (RevMan) version 5.4 [40] and R (version 4.4.1, [41]) using the *meta* [42] and *PRISMA2020* [43] packages.

## Results

### Description of included studies

**Fig. 1:**
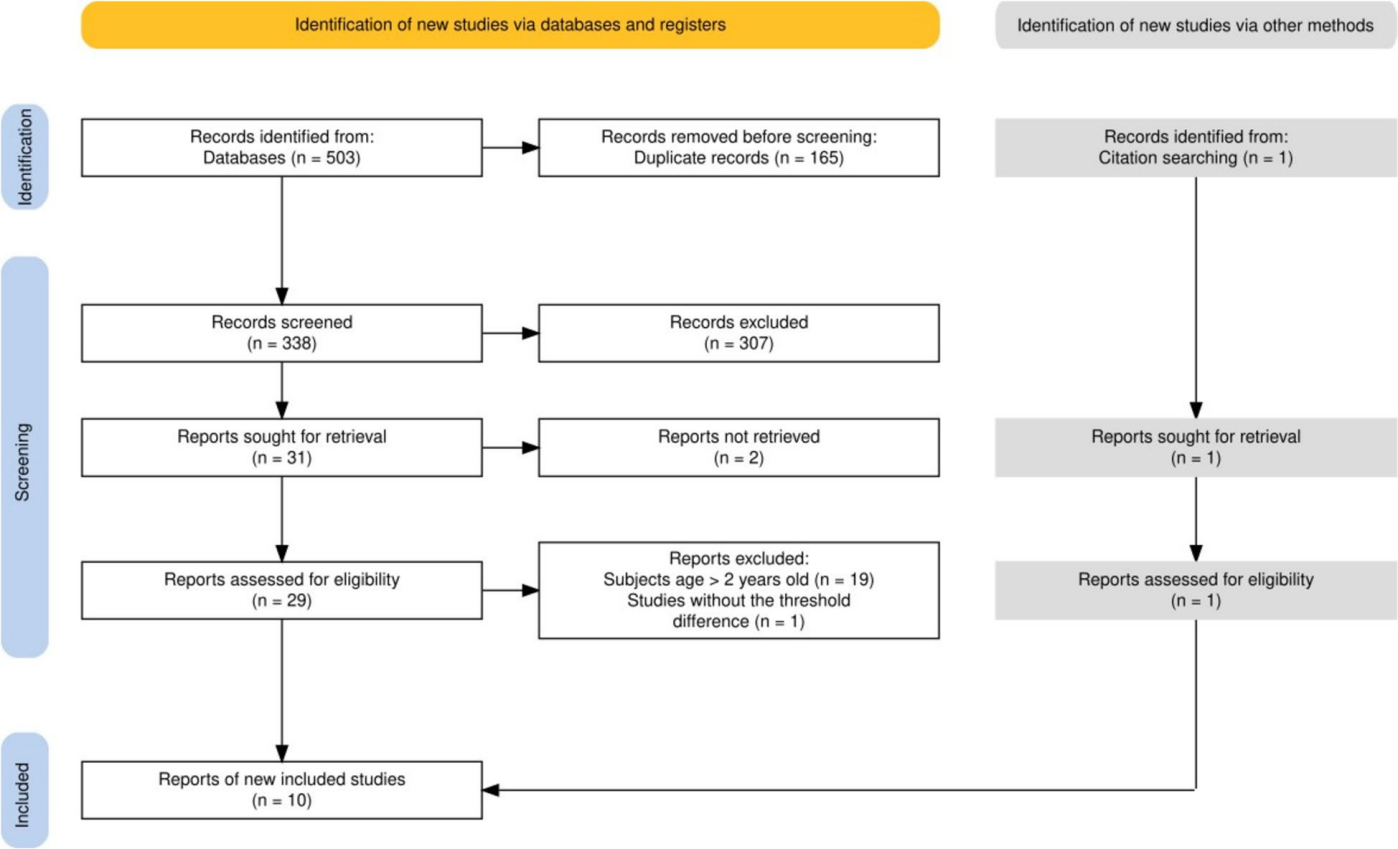
Flow chart of literature search results and study selection process

Figure 1 presents the flow chart as outlined in PRISMA 2020 [32]. Three hundred thirty-eight articles were identified in the initial searches after duplicates were removed; 25 studies were excluded because they did not concern infant subjects; 48 articles were removed because these studies did not perform BA, or were without the threshold differences data, or data did not allow calculation of threshold differences; 180 articles were excluded because their topics were not related to the comparison between ASSR thresholds and behavioural hearing thresholds; 26 articles were removed because they were published before 2000; 28 articles were excluded because they were not available in English; two were excluded because they did not have a full text (attempts to contact the authors were unsuccessful). Twenty-nine full-text articles remained and were reviewed for inclusion evaluation. Of these, 20 articles were removed: 19 were excluded because the results of these studies included participants who were older than 2 years of age, and one article was excluded because this study was without the data of threshold differences and it was not possible to calculate the threshold differences from the data presented in the article. One additional article was added through a manual search of the reference lists of included studies, but no new studies were found in the references of this additional article [21]. A total of 10 studies were included: Alaerts et al. (2010) [14], Casey & Small (2014) [15], Chou et al. (2012) [16], Cone-Wesson et al. (2002) [17], Luts et al. (2004) [2], Núñez-Batalla et al. (2016) [20], Rance & Briggs (2002) [21], Rance et al. (2005) [23], Savio et al. (2006) [24], Venail et al. (2015) [25].

### Quality assessment

**Fig. 2:**
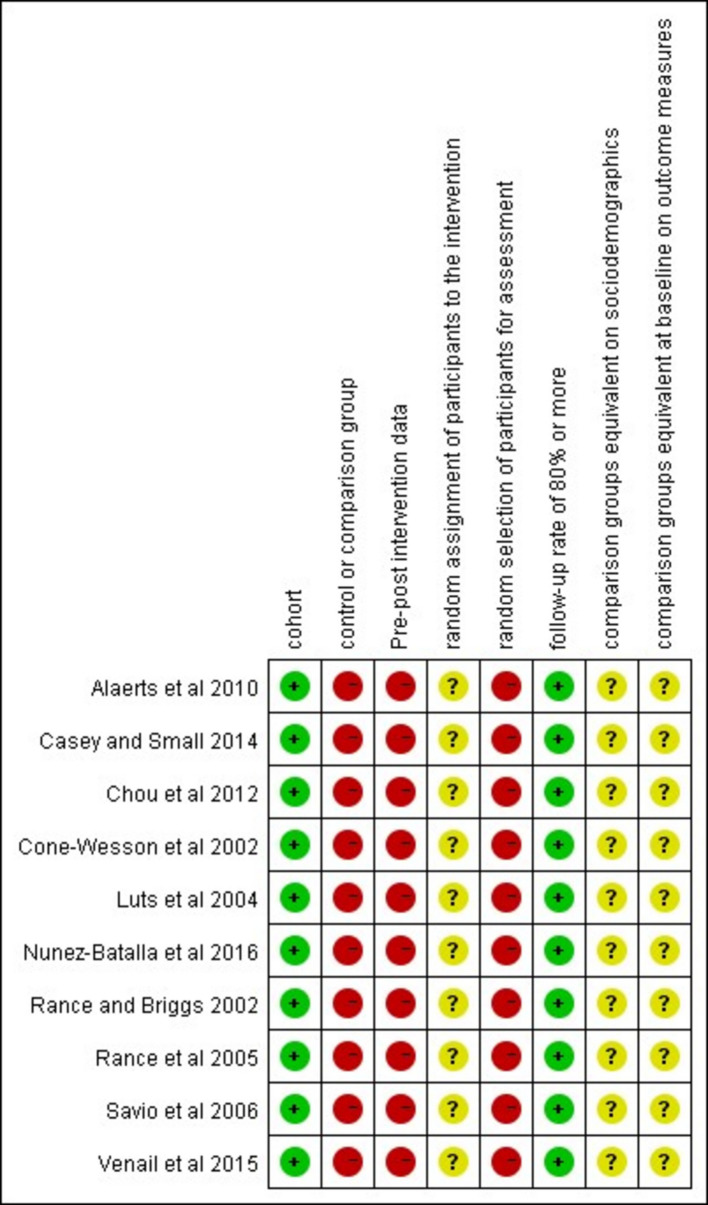
Assessment of bias for cohort studies according to the Evidence Project risk of bias tool [37]. “ + ” symbols in green mean “yes” and indicate the study fulfils the criterion. “-” symbols in red mean “no” and indicate the study does not fulfil the criterion. “?” symbols in yellow mean “not applicable” and indicate the criterion does not apply to the study

Figure 2 shows the assessment of the studies performed using the Evidence Project risk of bias tool [37]. The first three items summarise the study design and indicate that the studies included were cohort. Since these studies were within-subject designs, items related to comparison groups and random assignment of participants to the intervention were not applicable to them. All studies met the follow-up criteria, but none of these studies met the criteria of “random selection of participants for assessment” since they all set the inclusion criteria for participants [2, 14–17, 20, 21, 23–25]. The random selection of participants for assessment was relatively difficult to apply in these studies because ASSRs were often measured in children who were referred from the newborn hearing screening and the inclusion criteria aimed to avoid inclusion of conditions like retro-cochlear pathology [2, 14–17, 20, 21, 23–25].

The Evidence Project risk of bias tool was designed for both randomised and non-randomised study designs, but it inclines more towards between-subjects design, thus the Newcastle–Ottawa Quality Assessment Scale for cohort studies was additionally used [38].

Table 1 shows the assessment results according to the Newcastle–Ottawa tool [38]. A maximum of 9 stars can be awarded for a study in total. All studies included in the narrative summary acquired at least 5 stars. For the first part “Selection”, the subjects in these studies were selected and there were no non-exposed cohorts, thus they did not satisfy the item “representativeness of the exposed cohort” and the item “selection of the non-exposed cohort” did not apply to them. For the second part “Comparability”, the investigating factors included controlling for age and gender as the most important factors with additional factors of (1) Same use of sedative/anaesthesia for all subjects; (2) Same protocol of BA used in all subjects; (3) The exclusion of subjects with middle ear problems and retro-cochlear problems (like auditory neuropathy); (4) Without observer bias (audiologist who performed the behavioural audiometry had no knowledge of ASSR results). Only three studies [2, 20, 25] provided the gender information for subjects included in the calculation for threshold differences. Venail et al. (2015) [25] was awarded one star for the “Comparability” since the numbers of males and females were equal (i.e., 16). None of these studies controlled all the above-mentioned additional factors or provided information for them. For the part “Outcome”, all studies were awarded the maximum number of stars since the follow-up was long enough and adequate, and the outcomes were accurate with careful original medical records.

The studies had similar assessment results according to these two tools [37, 38]. All 10 studies passed the narrative summary and were defined as moderate-quality.

**Table 1:** Assessment of bias for cohort studies according to the Newcastle–Ottawa Quality Assessment form for cohort studies [38]. A maximum of 9 stars can be awarded for a study. A maximum of 4 stars can be given for Selection. Comparability can be awarded a maximum of 2 stars and for Outcome, the maximum number of stars is 3

Study	Selection	Comparability	Outcome	Total
Alaerts et al. (2010) [14]	**		***	5
Casey & Small (2014) [15]	**		***	5
Chou et al. (2012) [16]	**		***	5
Cone-Wesson et al. (2002) [17]	**		***	5
Luts et al. (2004) [2]	**		***	5
Núñez-Batalla et al. (2016) [20]	**		***	5
Rance & Briggs (2002) [21]	**		***	5
Rance et al. (2005) [23]	**		***	5
Savio et al. (2006) [24]	**		***	5
Venail et al. (2015) [25]	**	*	***	6

### Characteristics of studies included

Tables 2 and 3 show the characteristics of studies investigating the relationship between ASSR and BA. All ten studies were cohort and included 1268 participants in total. The sample size ranged from 7 to 556 subjects. The exact number for female and male subjects used for calculating threshold differences was given only in three articles: Núñez-Batalla et al. (2016) [20] (Male: 24; Female: 11), Venail et al. (2015) [25] (Male: 16; Female: 16), and Luts et al. (2004) [2] (Male: 1; Female: 6). The age of participants in the studies included was less than 2 years old (0.5 to 19.0 months of age). All studies used the 80-Hz ASSR except [20], which did not provide information about the modulation rate. Five studies used mixed-modulated tones as stimuli [2, 14, 17, 21, 23]. Two studies used amplitude-modulated tones [16, 24]. Exponential-envelope modulated tones and narrow-band CE-Chirp were the stimuli used by [15] and [25], respectively. For ASSR recording, multiple frequencies were recorded simultaneously binaurally in four studies [14, 16, 20, 25]. Multiple frequencies were recorded monaurally in two studies [15, 24]. Single frequency was recorded monaurally in three studies [17, 21, 23]. For the state of arousal in ASSR recording, the participants in [14, 15, 20, 21, 23] were all tested under natural sleep conditions; whereas the subjects in [2, 16] were all tested under the use of general anaesthesia. In [17, 24, 25] anaesthesia was used on a few subjects only.

**Table 2:** Characteristics of studies included. Hearing condition: HI, hearing-impaired subjects; NH, normal-hearing subjects; Combination, both hearing-impaired and normal-hearing subjects. Sample size: the number of subjects included in the calculation of threshold difference between ASSR and behavioural audiometry (BA); M, male; F, female; SNHL: Sensorineural hearing loss. Age: mo., months. “-” means the information was not found in the article

Study	Study design	Hearing condition	Sample size	Age	Exclusion of AN	Exclusion of middle ear disease	Observer bias
Alaerts et al. (2010) [14]	Cohort	Combination	50	< 5mo	-	Yes	Yes
Casey & Small (2014) [15]	Cohort	NH	12	6.5–19 mo	Yes	Yes	-
Chou et al. (2012) [16]	Cohort	Combination	216	1–13 mo	-	Yes	-
Cone-Wesson et al. (2002) [17]	Cohort	Combination	51	Mean = 16 mo	Yes	No	Yes
Luts et al. (2004) [2]	Cohort	HI	7 (M: 1; F: 6)	3–14 mo	-	-	-
Núñez-Batalla et al. (2016) [20]	Cohort	Combination	35 (M: 24; F: 11)	Mean = 4 mo	-	-	-
Rance & Briggs (2002) [21]	Cohort	HI	184	1–8 mo	Yes	Yes	-
Rance et al. (2005) [23]	Cohort	Combination	285 NH; 271 SNHL	0.5–3 mo	Yes	Yes	-
Savio et al. (2006) [24]	Cohort	Combination	125	4–5 mo	-	-	-
Venail et al. (2015) [25]	Cohort	HI	32 (M: 16; F: 16)	1–17 mo	No	Yes	-

**Table 3:** Characteristics of studies included. Stimuli: AM, amplitude-modulated tones; MM, mixed-modulated tones; AM2, exponential-envelope modulated tones; NB, narrow band. Stimulus condition: BMF, binaural multiple frequency carrier; MMF, monaural multiple frequency carrier; MSF, monaural single frequency carrier. Use of anaesthesia: No = every subject was tested without the use of anaesthesia; Yes = every subject was tested with the use of anaesthesia; Partly used = the anaesthesia was used in a few subjects only. Type of behavioural audiometry: VRA, visual reinforcement audiometry; PA, play audiometry; BOA, behavioural observation audiometry. “-” means the information was not found in the article

Study	ASSR				Behavioural audiometry		
	**Modulation Rate**	**Stimuli**	**Stimuli Condition**	**Use of anaesthesia**	**Type**	**Testing tone**	**Transducer**
Alaerts et al. (2010) [14]	80 Hz	MM (100%AM; 20%FM)	BMF	No	VRA/PA	Warble tone/Pure tone	Loudspeaker/headphone/insert phone
Casey & Small (2014) [15]	80 Hz	AM^2^	MMF	No	VRA	Warble tones	Insert phone
Chou et al. (2012) [16]	80 Hz	AM	BMF	Yes	PA	-	Headphone/insert phone
Cone-Wesson et al. (2002) [17]	80 Hz	MM (100%AM; 20%FM)	BMF	Yes	BOA/VRA	-	Insert phone/loudspeaker
Cone-Wesson et al. (2002) [17]	80 Hz	MM (100%AM; 10%FM)	MSF	Partly used	VRA/PA	Warble tones/narrow band noise	Loudspeaker/headphone
Luts et al. (2004) [2]	80 Hz	MM (100%AM; 10%FM)	MSF	No	VRA	Warble tones	Headphone/tube phone
Núñez-Batalla et al. (2016) [20]	-	-	BMF	No	VRA	-	-
Rance & Briggs (2002) [21]	80 Hz	MM (100%AM; 10%FM)	MSF	No	VRA	-	Headphone/tube phone
Savio et al. (2006) [24]	80 Hz	AM	MMF	Partly used	-	-	-
Venail et al. (2015) [25]	80 Hz	NB CE-Chirp	BMF	Partly used	VRA	Warble tones	Loudspeaker

Different types of BA (i.e., behavioural observation audiometry, visual reinforcement audiometry, play audiometry) for subjects according to the developmental age and medical status of the child were used [2, 14, 21]. Warble tones were used most frequently in BA in these studies [14, 15, 17, 23, 25]. Pure tone/narrowband noise was also used as an alternative [14, 17]. The studies used different transducers in BA (e.g., insert phone, headphone, tube phone, or loudspeaker).

### Threshold differences

The mean threshold differences extracted from the included studies are in Table 4. The number of ears at each frequency was presented in some articles and some were obtained via direct communication with the authors [15, 20, 23].

**Table 4:** Summary of mean threshold differences (ASSR threshold minus behavioural hearing threshold) for studies included. Diff = mean threshold difference; N = number of ears. “-” means the information was not found in the article. R = right ear, L = left ear. NH = Normal-hearing, HI = Hearing-impaired. Bolded text indicates studies included in the meta-analysis

Study	Level	0.5 kHz		1 kHz		2 kHz		4 kHz	
		**Diff**	**N**	**Diff**	**N**	**Diff**	**N**	**Diff**	**N**
Alaerts et al. (2010) [14]	dB SPL	17 ± 15	-	16 ± 12	-	12 ± 12	-	16 ± 12	-
**Casey & Small (2014)**^a^ [15]	dB HL	22.0 ± 6.3	10	17.5 ± 9.6	4	14.5 ± 11.3	11	19.1 ± 16.2	9
Chou et al. (2012) [16]	dB HL	20.2 ± 13.9	-	16.8 ± 10.1	-	16.1 ± 10.8	-	13.8 ± 10	-
Cone-Wesson et al. (2002) [17]	dB HL	−3.72 ± 15	-	−0.45 ± 14.7	-	1.67 ± 13.7	-	−0.96 ± 15	-
**Luts et al. (2004)** [2]	dB SPL	4 ± 14	5	4 ± 11	11	−2 ± 14	10	−1 ± 13	8
**Núñez-Batalla et al. (2016)**^**b**^ [20]	dB HL	2.2 ± 14.4	67	3.3 ± 15.8	67	3.7 ± 14.4	68	3.2 ± 15.3	67
**Rance & Briggs (2002)**^**c**^ [21]	dB HL	6.3 ± 9.2	160	5.5 ± 7.2	232	4.0 ± 8.1	125	3.4 ± 11.1	131
**Rance et al. (2005) (HI)**^d^ [23]	dB HL	-	-	6.7 ± 12.1	506	-	-	14.9 ± 15.1	842
**Savio et al. (2006)** [24]	dB HL	15 ± 13	96	15 ± 13	96	13 ± 14	96	13 ± 15	96
**Venail et al. (2015)** [25]	dB HL	4.1 ± 11.3	32	1.8 ± 9.2	32	3.8 ± 8.3	32	0.5 ± 12.4	32

^a^Personal communication with Susan A. Small (21/07/2020)

^b^Personal communication with Faustino Núñez-Batalla (22/07/2020)

^c^Mean threshold differences included only ears with sensorineural hearing loss

^d^Personal communication with Gary Rance (20/07/2020)

**Fig. 3:**
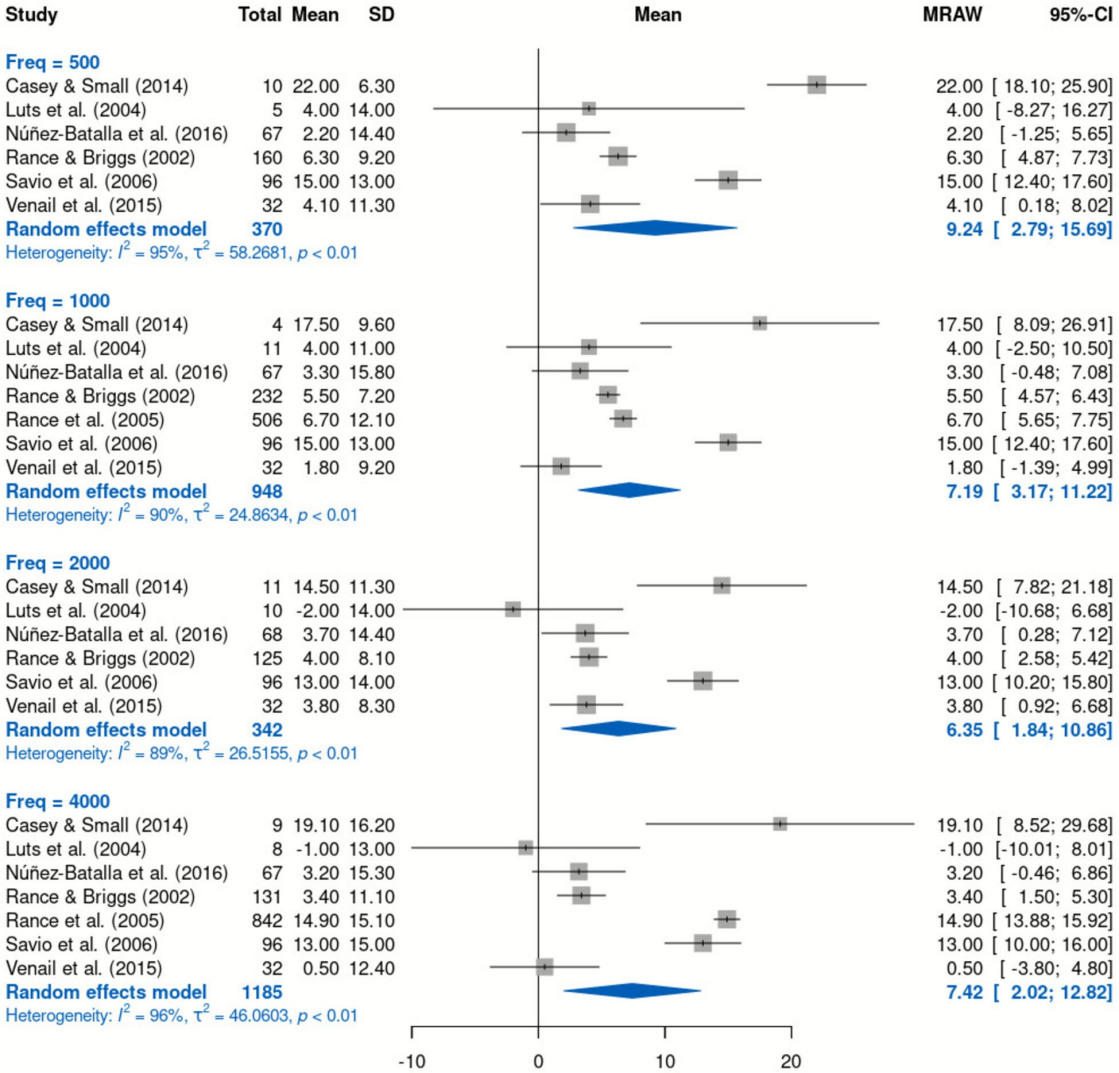
Random-effects model for the threshold differences between ASSR and BA at 0.5, 1, 2 and 4 kHz

The number of ears (i.e., number of ears for which the ASSR thresholds were measured) at each frequency used for the calculation of the threshold difference could not be obtained in 3 studies (Alaerts et al., 2010 [14]; Chou et al., 2012 [16]; Cone-Wesson et al., 2002 [17]). Therefore, only the 7 studies that provided the number of ears for the threshold difference at different frequencies were included in the calculation for the overall mean, SD, SE, and 95% CI [2, 15, 20, 21, 23, 25]. The studies showed high heterogeneity; therefore, a random effects model was applied to analyse the threshold differences for each frequency (Fig. 3). The mean threshold differences (± 95% confidence interval) between ASSR and BA were below 10 dB, i.e., 9.24 dB (± 6.45), 7.19 dB (± 4.02), 6.35 dB (± 4.51), and 7.42 dB (± 5.40), at 0.5, 1, 2, and 4 kHz, respectively.

## Discussion

The aim of the current study was to provide “correction” values for ASSR thresholds to estimate behavioural hearing thresholds in infants under 2 years of age. Here, we compare the found values with those reported in the literature, focusing on both variability and absolute values as a means of understanding differences in the results. A 10 dB difference in estimating the threshold can determine whether the infant requires intervention. Variability of 10 dB, however, implies considerable uncertainty in clinical decision-making.

In this meta-analysis, the overall mean difference between ASSR and BA was 7.55 dB, compared with 18.73 dB reported in [15], yielding a difference of 10.72 dB between the two studies (see Supplementary Table S1 for specific frequency values). Such a large correction value for estimating BA hearing thresholds could lead to either a false positive (in the case of overestimation of thresholds) or a false negative (in the case of underestimation) diagnosis. Thus, it is paramount that the correction values are “correct”.

However, the ASSR-BA threshold differences in [15] were based on data obtained only from normal-hearing infants. These values were larger and more variable than the threshold differences for combined group of normal-hearing and hearing-impaired infants in this meta-analysis. Therefore, using only a single group of infants may lead to inaccurate correction values. The lower variability in our data compared to [15] appears to be driven by the low variability in hearing-impaired thresholds, but we were unable to test this assertion due to lack of access to disaggregated data.

The smaller difference and variability of ASSR-BA thresholds in hearing-impaired subjects compared with normal-hearing subjects is supported by several studies both in adults and children [16, 44]. The amplitude of the ASSR generally increases with stimulus intensity. However, when the intensity is just above the hearing threshold, the amplitude increases more rapidly in hearing-impaired subjects than in normal-hearing subjects, a phenomenon known as “electrophysiological recruitment” [45]. Electrophysiological recruitment increases the amplitude of near-threshold responses in hearing-impaired subjects, making their responses easier to be detected and possibly explaining the better performance of ASSR in hearing-impaired subjects [45].

Another possible explanation could be that the ASSRs are not tested below 20 or 30 dB HL in normal-hearing individuals, whose behavioural thresholds could be lower than the minimum testing intensity of ASSR, which in turn increases the threshold difference between ASSR and BA and relatively makes ASSR perform worse in normal-hearing subjects compared to the hearing-impaired subjects [20, 24].

Compared with the threshold differences between ASSR and BA in subjects older than 6 years from the meta-analysis of Tlumak et al. (2007) [29], the ASSR-BA threshold differences in infants (< 2 years old) in the present study were on average 4.6 dB lower (see Supplementary Table S2). There are a number of possible explanations for the relatively lower ASSR-BA threshold differences in infants than in adults. First, pure-tone audiometry was used with older children and adults whereas infants were tested using BA [29]. The BA thresholds obtained in infants are more likely to correspond to the minimum response level, which is higher than hearing threshold in pure-tone audiometry [19]. ASSR thresholds are often higher than behavioural thresholds, thus, this may lead to smaller differences between ASSR thresholds and behavioural hearing thresholds in infants compared to adults and older children. Second, there was a time interval between ASSR and BA in infants during which the hearing might have changed [23]. If the hearing deteriorated during this time interval, this would result in a reduction in the threshold difference between ASSR and BA. Third, the ear canal volume of the infant was growing during this time interval between ASSR and BA, thus the sound pressure level (SPL) of the acoustic stimuli at the eardrum might decrease with time, also giving rise to a reduction of the threshold difference [19]. Fourth, infants were required to sleep during ASSR recording and most of them were given sedatives, but older children and adults just relaxed or were only encouraged to sleep during the test, therefore the noise level during the ASSR test might have been lower for infants resulting in better-quality recording and might have improved the threshold estimation with ASSR in infants, leading to smaller threshold differences between ASSR and BA in infants than in adults [29]. Luts et al. (2006) [19] suggested that taking the possible change of hearing during the time interval between ASSR and BA and the growth of ear canal volume of infants into account when using ASSR to predict hearing for infants could lead to a more accurate hearing-threshold estimation.

The larger threshold differences between ASSR and BA at 0.5 kHz than at 1, 2, 4 kHz have been reported previously [23, 26]. In this meta-analysis, the overall mean threshold difference was also found to be higher at 0.5 kHz than at other frequencies, as well as larger 95% confidence interval, indicating that ASSR method performed worse at 0.5 kHz than at other test frequencies. A possible explanation is that most environmental noises are dominated by low-frequency components, while the synchronization of the hair cells is better in the base of the cochlea than at the apex. Thus, the resulting signal-to-noise ratio is higher in the high-frequency range than in the low-frequency range, resulting in greater neural synchronization of potentials at high frequencies than at low frequencies [18].

There is significant heterogeneity among the studies included in the meta-analysis. This was probably due to disparities in infant hearing – some studies tested only normal-hearing infants, others only hearing-impaired infants, or a combination of both groups – and in the parameters of ASSR and BA across studies (see Table 3); for example, the BA (type, test tones, and transducers) and/or the use of anaesthesia differed in subjects in some individual studies [2, 21, 23–25]. The large heterogeneity may undermine the accuracy of the “correction” values. Future work should compare ASSR and BA thresholds in a large cohort of infants with a range of hearing thresholds and evaluate pooled sensitivity, specificity, and likelihood ratios.

The variability in threshold differences between ABR and BA is lower [3] or comparable [46] to that observed for ASSR. However, the ASSR ability to measure severe-to-profound hearing loss and record multiple frequencies in both ears, reducing testing time, enhances its clinical value. It can be used, for instance, to corroborate ABR results when ABR waves could lead to misinterpretation, in cases of permanent childhood hearing impairment, and in theatre settings, where threshold estimation using ABR can be challenging due to interference [47].

## Conclusions

Seven moderate-quality papers with large heterogeneity were included in the meta-analysis. On average, ASSR thresholds were 7 dB higher than behavioural thresholds. The “correction” values as a function of frequency were calculated and discussed. The variability of the “correction” values highlights the need for more and better studies comparing ASSR thresholds and behavioural hearing thresholds in infants younger than 2 years, to determine the optimal ASSR procedure for estimating hearing thresholds in infants.

## Supplementary Information

Supplementary Material 1.

Supplementary Material 2.

## Data Availability

All data generated or analyses during this study are included in this published article and its supplementary table.
