# Supplementary material for: The relationship between auditory steady-state response and behavioural audiometry in hearing estimation for infants: a meta-analysis
Source: Syst Rev. 2025 Dec 3;15:2. doi: 10.1186/s13643-025-03003-x (PMC12781307; doi:10.1186/s13643-025-03003-x)
Supplement: Supplementary file 1 — Supplementary Material 1. [file 13643_2025_3003_MOESM1_ESM.docx]

Supplemental material to:

**The relationship between auditory steady-state response and behavioural audiometry in hearing estimation for hearing-impaired infants: a meta-analysis**

Xin Huang^1^, Karolina Kluk^1^, & Emanuele Perugia^1^

^1^Manchester Centre for Audiology and Deafness (ManCAD), School of Health Sciences, Faculty of Biology, Medicine and Health, University of Manchester, UK

Corresponding author:

Dr Emanuele Perugia

emanuele.perugia@manchester.ac.uk

**Table S1**. The comparisons between the threshold differences for normal-hearing subjects in Casey & Small (2014) and the differences for infants in the present meta-analysis.

| Study | Age  (years) | Hearing | Test | Threshold differences (mean ± 95 confident interval) | | | |
| --- | --- | --- | --- | --- | --- | --- | --- |
|  |  |  |  | 0.5 kHz | 1 kHz | 2 kHz | 4 kHz |
| Casey & Small  (2014) | <2 | Normal | 80Hz ASSR | 22.0 ± 3.90 | 17.5 ± 9.41 | 14.5 ± 6.68 | 19.1 ± 10.58 |
| Present | <2 | Combination | 80Hz ASSR | 9.24 ± 6.45 | 7.19 ± 4.02 | 6.35 ± 4.51 | 7.42 ± 5.40 |

**Table S2**. The comparisons between the threshold differences for subjects in Tulumak et al. (2007) and the differences for infants in the present meta-analysis.

| Study | Age  (years) | Hearing | Test | Threshold differences (mean ± 95 confident interval) | | | |
| --- | --- | --- | --- | --- | --- | --- | --- |
|  |  |  |  | 0.5 kHz | 1 kHz | 2 kHz | 4 kHz |
| Tlumak et al.  (2007) | >6 | Normal | 80Hz ASSR | 16.51 ± 1.25 | 12.97 ± 1.22 | 10.76 ± 1.01 | 15.09 ± 1.1 |
|  |  | Impaired | 80Hz ASSR | 14.46 ± 1.34 | 10.23 ± 1.29 | 8.57 ± 1.22 | 8.30 ± 1.31 |
| Present | <2 | Combination | 80Hz ASSR | 9.24 ± 6.45 | 7.19 ± 4.02 | 6.35 ± 4.51 | 7.42 ± 5.40 |
